# Scrolling for fun or to cope? Associations between social media motives and social media disorder symptoms in adolescents and young adults

**DOI:** 10.3389/fpsyg.2024.1437109

**Published:** 2024-08-02

**Authors:** Lisa B. Thorell, Milena Autenrieth, Alice Riccardi, Jonas Burén, Sissela B. Nutley

**Affiliations:** ^1^Department of Clinical Neuroscience, Karolinska Institutet, Stockholm, Sweden; ^2^Department of Psychology, Renzo Canestrari, University of Bologna, Bologna, Italy; ^3^Department of Psychology, University of Gothenburg, Gothenburg, Sweden

**Keywords:** social media, addiction, motives, social media disorder, escape, social compensation

## Abstract

**Introduction:**

Although not yet recognized as an official disorder, Social Media Disorder (SMD) has recently received considerable interest in the research. However, relatively little is known about underlying motives for social media use and to what extent motives show differential associations with SMD symptom severity and SMD diagnosis. The overall aim of the present study was therefore to examine motives for social media use in relation to (1) which motives are most common, (2) associations between motives and both SMD symptom severity and SMD diagnosis, and (3) the effects of sex and age.

**Methods:**

Data were collected through a digital survey (*n* = 1820) and included both high school students (*n* = 924) and university students (*n* = 896). Six different motives were assessed, and SMD was measured in relation to both Heavy Involvement and Negative Consequences of social media use.

**Results:**

The results showed that the most common social media use motives were Entertainment, Social Maintenance, and Information and Skills. However, it was the three least common motives – Social Compensation, Self-status, and Escape – that were most strongly associated with SMD symptom severity and SMD diagnosis. These three motives explained as much as 42% of the variance in negative consequences of social media use. Only a few small effects of sex or age were found.

**Discussion:**

Some social media use motives are much more strongly associated with SMD than others are. This could indicate that prevention and intervention programs should target these motives specifically, rather than focusing on social media use in general.

## 1 Introduction

Social Media Disorder (SMD) has not yet been recognized as an official disorder within any diagnostical system. However, it has been suggested (e.g., van den Eijnden et al., 2016; van Rooij et al., 2017; Reer et al., 2021) that the same criteria used to define Internet Gaming Disorder (IGD) in the 5th version of the Diagnostical and Statistical Manual of Mental Disorders (DSM-5; American Psychiatric Association, 2013) can also be used to define SMD, by replacing the word ‘gaming’ with ‘social media.’ When using these or similar criteria, the prevalence of SMD (≈ 5%; Cheng et al., 2021) has been shown to be higher than that of IGD (≈ 2%; Stevens et al., 2021), and several studies have drawn parallels between the psychosocial consequences (e.g., anxiety, depression, social problems) of addictive gaming and addictive use of social media (e.g., Andreassen et al., 2016; Pontes, 2017; Wong et al., 2020). Thus, previous research suggests that SMD is a serious condition, but more research is needed to better understand how it should be defined and how to best provide support. In a review of theories and models applied in studies of social media addiction (Sun and Zhang, 2021), motives for social media use were identified to be of major importance. As described in more detail below, many studies have been conducted with the aim of identifying different motives for social media use, but relatively few studies have investigated the link between different social media use motives and SMD symptom severity. Given that the motives for social media use could define how individuals engage with social media, a more in-depth understanding of the link between motives and addictive use of social media should be of importance both for identifying individuals at risk of developing SMD and for developing effective prevention and intervention programs. The overall aim of the present study was therefore to investigate to what extent motives for social media use are associated with SMD symptom severity and SMD diagnosis and to what extent age and sex moderate these associations.

### 1.1 Social media use motives

Previous studies have varied greatly regarding the number of social media use motives identified. Some of these studies (e.g., Senkbeil, 2018) have also used a broader scope than social media (e.g., internet use in general), whereas others (e.g., Marino et al., 2018) have used a narrower scope (e.g., investigating motives for use of one specific social media platform such as Facebook, Instagram, TikTok, or X, formerly Twitter) or only focused on some specific aspects of social media use motives (e.g., Rodgers et al., 2021). When combining information from previous research focusing specifically on social media, the following motives are most often included: social connection, information seeking, entertainment, escapism, and self-expression/self-status (Floros and Siomos, 2013; Süral et al., 2019; Kircaburun et al., 2020; Schivinski et al., 2020; Stockdale and Coyne, 2020; Jarman et al., 2021; Rodgers et al., 2021; Arness and Ollis, 2022). Among these motives, the motives receiving the highest scores (i.e., reported being used most frequently) have varied across studies and have included social connection (e.g., Brailovskaia et al., 2020; Schivinski et al., 2020; Rodgers et al., 2021), information seeking (e.g., Brailovskaia et al., 2020; Kircaburun et al., 2020) and entertainment (Alhabash and Ma, 2017; Süral et al., 2019). However, as further described below, the operationalization of these five motives has varied substantially across studies, and the most common motives often show the highest associations with addictive social media use.

With regard to social connection, two different theoretical models have been presented: Social Enhancement Theory (McKenna et al., 2002) and the Social Compensation Hypothesis (Valkenburg et al., 2005). The former assumes that people with high levels of social skills use social media to improve their social connections with already established friends, whereas the latter proposes that people with poor social skills use social media to find new friends, most often because they have difficulties finding friends in real life. Despite this, many previous studies investigating social media use motives have asked about socialization in general or only one aspect of social communication rather than distinguishing between maintaining existing friendships and using social media to compensate for difficulties finding offline friends (e.g., Stockdale and Coyne, 2020; Jarman et al., 2021; Rodgers et al., 2021). The importance of making this distinction has been illustrated by the finding that addictive use of social media appears to be more strongly associated with using social media to find new friends compared to using social media to maintain offline friendships (Süral et al., 2019; Kircaburun et al., 2020).

Regarding information as a motive for using social media, it should be noted that most studies (e.g., Süral et al., 2019; Kircaburun et al., 2020; Stockdale and Coyne, 2020) have defined this as information seeking (e.g., finding information and learning new skills), whereas a few studies (e.g., Jarman et al., 2021) have instead focused on information sharing (i.e., uploading information about oneself on social media). Most studies investigating information sharing have, however, used this to capture aspects related to showcasing an idealized self, gaining popularity and establishing self-status (e.g., Süral et al., 2019; Kircaburun et al., 2020; Omar and Dequan, 2020). Regarding associations with addictive use of social media, associations have generally been found with information sharing (Schivinski et al., 2020; Tang et al., 2022) and self-status (Süral et al., 2019; Kircaburun et al., 2020), but not with information seeking (Süral et al., 2019; Brailovskaia et al., 2020; Kircaburun et al., 2020; Ponnusamy et al., 2020; Stockdale and Coyne, 2020; Arness and Ollis, 2022).

Previous studies of gaming (e.g., Kuss et al., 2012; Bäcklund et al., 2022) have shown that addictive use is most strongly related to the motive escape (i.e., using computers games to escape reality or avoid negative feelings), which is hardly surprising considering that escape is listed as one of the criteria for Internet Gaming Disorder (IGD; American Psychiatric Association, 2013). Using internet to escape real life problems or to alleviate dysphoric moods has a central part in both the model for compensatory internet use (Kardefelt-Winther, 2014) and the Interaction of Person-Affect-Cognition-Execution (I-PACE) model (Brand et al., 2016). Although escape may help the individual to temporarily cope, it will not address the source of the problem and could therefore maintain, or even increase, addictive internet use over time. The few studies investigating social media addiction and the motive escape have found that this motive shows a stronger association with addictive use compared to other motives (Brailovskaia et al., 2020; Schivinski et al., 2020). However, it should be noted that escape is not only an important motive for using social media, but also one criterion for SMD. Thus, it should be important to determine whether the motive escape is as strongly associated with addictive use of social media when this overlap is excluded.

Finally, it should be noted that entertainment as a motive for using social media often includes aspects that are not necessarily related to social media being fun, but to social media use for relaxation (Schivinski et al., 2020; Arness and Ollis, 2022), passing the time (Süral et al., 2019; Kircaburun et al., 2020; Schivinski et al., 2020; Jarman et al., 2021), and even overcoming boredom (Stockdale and Coyne, 2020; Jarman et al., 2021). Regarding associations between the motive entertainment and addictive use of social media, previous studies have shown mixed results, with some studies finding associations (Süral et al., 2019; Kircaburun et al., 2020) and others failing to do so (Ponnusamy et al., 2020; Arness and Ollis, 2022). The reason for this inconsistency across studies could perhaps be explained by differences in how addictive use has been operationalized. It has been argued (e.g., Wichstrøm et al., 2019; Burén et al., 2023a) that it is important to distinguish between the SMD symptom criteria related to heavy involvement (e.g., preoccupation, withdrawal symptoms) and those related to negative consequences (e.g., prioritizing social media instead of social contacts/work/school or getting into conflicts). However, no previous studies have investigated social media use motives in relation to these two facets of social media addiction.

### 1.2 Age and sex as moderators

Previous research has found that SMD is more common among females (4.1%) than males (2.9%; Boer et al., 2021), and more common among university students (4.0%) than adolescents (2.6%; Burén et al., 2023a). Regarding social media use motives, previous research has identified only small to negligible effects of age (Brailovskaia et al., 2020; Kircaburun et al., 2020; Stockdale and Coyne, 2020). However, most previous studies have only investigated adult samples, with a predominance of university students and individuals in early adulthood. It is therefore not known whether the motives for using social media differ between adolescents and adults.

As for sex differences in motives for using social media, prior research has highlighted small and somewhat heterogeneous effects. In general, females tend to prefer using social media for maintaining existing social relations, whereas males use social media more for meeting new people or for network expansion (Horzum, 2016; Kircaburun et al., 2020; Schivinski et al., 2020; Stockdale and Coyne, 2020). Beyond these findings, it is worth noting that additional sex differences have been found such as higher levels in females compared to males with regard to self-presentation (Horzum, 2016), boredom relief (Brailovskaia et al., 2020; Stockdale and Coyne, 2020), as well as task management and information/educational purposes (Horzum, 2016; Kircaburun et al., 2020). However, these effects have been either small or only found in some studies and not others. To our knowledge, previous studies have only investigated main effects of age and sex and not used age or sex as moderators regarding the association between social media motives and social media addiction. The exception is the study by Stockdale and Coyne (2020), who investigated social media motives at age 17–19 years in relation to problematic mobile phone use 2 years later and found that associations did not differ significantly between males and females. In summary, there appears to be some sex differences with regard to social media motives, primarily with regard to whether social media is used to maintain existing relations or to create new relations. We still know very little about whether the associations between social media use motives and SMD symptom severity are stronger for males or for females.

### 1.3 Aim of the present study

As described above, social media use motives have been investigated in several previous studies. However, several important aspects have not yet been addressed. First, only very few studies have investigated associations between social media use motives and SMD symptom severity. In the few studies addressing this issue, SMD symptom severity has not been operationalized based on specific criteria (e.g., DSM-5; American Psychiatric Association, 2013) and a distinction has not been made between heavy involvement and negative consequences in social media. Second, we do not know to what extent some social media use motives are associated with an increased risk of meeting the criteria for SMD (i.e., ≥5 symptom criteria met). Third, age and sex have not been examined as possible moderators. Fourth, the number of motives investigated in any given study has been limited, which means we do not know how much of the variance in SMD symptom severity can be explained by social media use motives. Finally, several studies have investigated broad motivational categories rather than more specific motives (e.g., intrapersonal motives in general, internal/external motives, or failing to distinguish between different types of social motives). This could be problematic, as these broad categories make it difficult to determine which aspects are actually related to addictive social media use. To add new information to this research field, the present study aimed to address the following specific research questions:

1. What are the most common social media use motives among adolescents and young adults, and are there age and sex differences with regard to these motives?
2. Are the social media use motives associated with SMD symptom severity and increased risk of meeting the symptom criteria for SMD?
3. Are there age and sex differences in the proportion meeting the symptom criteria for SMD, and are there moderating effects of age and sex regarding the association between social media use motives and SMD symptom severity?

## 2 Method

### 2.1 Participants and procedure

A total of 1820 social media users (62.6% females) were included in the study, of whom 924 (60.9% females) were high school students and 896 (64.3% females) university students. The mean age of the total sample was 20.37 years (*SD* = 4.33), with a mean age of 17.19 (*SD* = 1.03) years for high school students and 23.67 years (*SD* = 3.96) years for university students. See Table 1 for further descriptive data. The participants were asked to complete a digital survey, and the data were collected at five high schools and seven universities in various parts of Sweden. High school students were recruited via contact with principals at randomly selected high schools and the students completed the survey individually on a computer or mobile device in the classroom under the supervision of a teacher. University students were recruited by research assistants who visited the seven universities and approached students on campus to ask if they were willing to participate in the study. The university students completed the survey on a mobile device on campus, between classes. Informed consent was collected from all participants, and they were instructed that they could withdraw from the study at any time without providing a reason for doing so. In accordance with the regulations established by the Swedish Ethical Review Authority, parental consent was not needed given that all participants were at least 15 years old. The study was carried out in accordance with the Code of Ethics of the World Medical Association (Declaration of Helsinki) for experiments involving humans, as well as institutional requirements.

**Table 1:** Descriptive data.

	High school		University	
	Females (*n* = 563)	Males (*n* = 361)	Females (*n* = 576)	Males (*n* = 320)
Age, mean (SD)	17.17 (1.04)	17.22 (1.01)	23.43 (3.93)	24.05 (3.90)
Mother’s education (%)				
Mandatory schooling (≤9 years)	2.1	3.6	4.3	5.3
High school	22.4	18.6	24.5	24.4
University	70.5	65.8	69.6	68.4
Do not know	5.0	11.9	1.6	1.6
Father’s education (%)				
Mandatory schooling (≤ 9 years)	6.7	8.0	4.9	4.7
High school	25.9	31.9	32.9	29.7
University	60.4	48.2	59.8	59.7
Do not know	6.7	11.9	2.4	5.6
Ethnic background (%)				
Sweden	88.6	87.0	74.7	76.4
Europe	4.8	4.2	16.3	13.2
Outside Europe	6.6	8.8	8.7	9.7
Missing answer	0.0	0.0	0.3	0.6
Three most often used apps (%)^a^				
Snapchat	85.8	80.9	49.3	41.6
Instagram	85.8	68.7	90.4	75.3
TikTok	78.7	60.7	48.1	22.2
Facebook	0.3	6.4	33.7	40.6
YouTube	16.7	36.0	23.6	32.5

^a^ Only social media apps which were often used by at least 10% within one group are reported.

### 2.2 Measures

#### 2.2.1 Social media use motives

As part of the present study, we generated 12 items, two items for each of the six different social media use motives identified as being of most importance in previous research. All items were answered on a 5-point Likert scale ranging from 1 = *Definitely not true* to 5 = *Definitely true*. The following six motives were included: (1) Social Maintenance (e.g., “…because I want to keep in contact with friends I also see in real life”); (2) Social Compensation (e.g., “...because I think it’s easier to connect with people digitally compared to in real life”); (3) Information & Skills (e.g., “...to search for information [e.g., interests, solution to problems”]); (4) Entertainment (e.g., “...because it’s entertaining/fun”); (5) Escape (e.g., “...to escape from the real world for a while”); (6) Self-status (e.g., “...because social media are an important way for me to gain high status among my peers”). A Principal Component Analysis (PCA), with a Direct Oblimin rotation method, was performed to assess the factorial validity of the items. The Kaiser’s-Meyer-Olkin measure of sampling adequacy was 0.71 and Bartlett’s test of sphericity was significant (<0.001), indicating that the items were suitable for PCA (Hutcheson and Sofroniou, 1999; Field, 2013). Parallel analysis indicated that six factors should be retained, and the PCA showed that a 6-factor solution resulted in six, clearly interpretable factors with no cross-loadings; the factor loadings ranged from 0.55 to 0.93. For each factor, we calculated the mean of the two items for each scale, with higher scores indicating greater motivation. In addition, we classified the motives into the following three categories: low levels (i.e., mean value = 1.0), medium levels (i.e., mean value >1.0 but <4) and high levels (mean value ≥4.0).

#### 2.2.2 SMD symptom severity and SMD diagnosis

To assess SMD, we used the Gaming and Social Media Questionnaire (GSMQ-9; Burén et al., 2023a). The GSMQ-9 consists of nine items, which correspond to the nine symptom criteria presented in the DSM-5 (American Psychiatric Association, 2013) for Internet Gaming Disorder, although we replaced the word ‘gaming’ with ‘social media.’ The GSMQ-9 includes two subscales, with four items measuring Heavy Involvement (i.e., preoccupation, withdrawal, tolerance, and unsuccessful attempts to control), and five items measuring Negative Consequences (i.e., loss of interest, continued excessive use, deception, escape, jeopardizing career/relationship). Each item is rated on a 5-point Likert scale, ranging from 0 (strongly disagree) to 4 (strongly agree), and we used the mean value for each subscale in the analyses. Participants were considered to meet a symptom criterion if they scored ≥3 on the 5-point scale, and in line with the DSM-5 criteria (American Psychiatric Association, 2013), they were considered to meet the symptom criteria for SMD if they had ≥5 symptoms. Previous research has shown that the GSMQ-9 has good psychometric properties regarding factor structure, test–retest reliability, and internal consistency (Burén et al., 2023a). For the present study, internal consistency for the two subconstructs remained acceptable for both adolescents (Heavy Involvement: *α* = 0.79; *ω* = 0.80; Negative Consequences: *α* = 0.72; *ω* = 0.73) and university students (Heavy Involvement: *α* = 0.80; *ω* = 0.80; Negative Consequences: *α* = 0.77; *ω* = 0.79).

### 2.3 Statistical analyses

Missing data were handled using listwise deletion, which was considered appropriate given that missingness was under 5%. For each variable containing outliers, the outlier labeling rule was applied by multiplying the interquartile range by a factor of 2.2 to adjust the outlier scores (Iglewicz and Hoaglin, 1993). As addictive social media use can be regarded both as a dimension (i.e., from low to high symptom levels) and as a category (i.e., meeting or not meeting the symptom criteria for SMD), we included both dimensional and categorical analyses for most of the research questions.

For the first research question, we calculated the mean values for the different motives and used 2-way factorial analyses of variances (ANOVAs) to investigate main and interaction effects of age group and sex. To determine the strength of each effect, we used partial *η*^2^, with a value of 0.01 indicating a small effect, a value of 0.06 indicating a medium effect, and a value of 0.14 indicating a large effect.

Regarding the second research question, we first used Pearson’s bivariate correlation analyses to examine the association between social media use motives and SMD symptom severity, with separate correlations for Heavy Involvement and Negative Consequences. Given the large sample size, we interpreted coefficients of at least medium effects sizes (i.e., *r* ≥ 0.30) as meaningful, rather than focusing on statistical significance (i.e., *p* < 0.05). Thereafter, linear regression analyses were used to assess the independent contribution of each social media use motive and the explained variance for Heavy Involvement and Negative Consequences. The Variance Inflation Factors (VIF) were all below 1.79, indicating no issues with multicollinearity. Finally, we used logistic regression analyses to examine to what extent individuals with high levels of a specific social media use motive were at increased risk of meeting the symptom criteria for SMD compared to those with low or medium levels.

Concerning the third research question, chi-square analyses were first used to examine age and sex differences in the proportions of individuals meeting the symptom criteria for SMD. Effect sizes were assessed using Cramer’s *V,* with a value of 0.10 indicating a small effect, a value of 0.30 indicating a medium effect, and a value of 0.50 indicating a large effect. Next, we used regression analyses to investigate moderating effects of age and sex on the association between social media use motives and SMD symptom severity (i.e., Heavy Involvement or Negative Consequences). After standardizing the variables, each one of the motives and either sex or age were entered in the first step and the interaction term in the second step.

## 3 Results

### 3.1 Which social media use motives are most common?

As seen in Table 2, the motives Entertainment, Information and Skills, and Social Maintenance had the highest mean value across both age groups and for both males and females. The two motives with the lowest mean values were Social Compensation and Self-status. For all motives except Social Compensation, females had significantly higher mean values than males, with small effects sizes for all motives, except for Escape (*η*^2^ = 0.08), which had a medium effect size. Regarding differences between the two age groups, three significant effects were found, with university students scoring significantly higher than high school students for Information and Skills, Escape, and Self-status. However, all effects sizes for age were small. Finally, four significant interaction effects were found. For the motive Social Compensation, university students scored higher than high school students among males, whereas the opposite pattern was found for females. For the Information and Skills motive, university students scored higher than high school students among females, whereas only small age differences were found among males. For the Escape motive, university males reported higher values than high school males, whereas there were small age differences for females. Finally, for the motive Self-status, university students scored higher than high school students among males, whereas the difference between the two age groups were negligible for females. However, it is important to consider that the effect sizes for the significant interactions were all small.

**Table 2:** Means and standard deviations for social media use motives among high school and university students, and males and females.

	High school		University		Two-way ANOVA		
	Females (n = 563)	Males (n = 361)	Females (n = 576)	Males (n = 320)	*F* (age) (partial *η^2^*)	*F*(sex) (partial *η^2^*)	*F* (age x sex) (partial *η^2^*)
Motives, mean (SD)							
Social maintenance	3.46 (0.84)	3.11 (0.90)	3.44 (0.84)	3.14 (0.89)	0.01 (0.00)	53.13 (0.03)***	0.24 (0.00)
Social Compensation	1.86 (0.88)	1.74 (0.78)	1.74 (0.87)	1.70 (0.85)	3.11 (0.00)	3.39 (0.00)	1.05 (0.00)
Information and skills	3.61 (0.84)	3.62 (0.91)	3.87 (0.72)	3.59 (0.98)	6.78 (0.00)**	8.70 (0.01)**	9.94 (0.01)**
Entertainment	4.09 (0.67)	3.83 (0.81)	3.99 (0.72)	3.80 (0.75)	2.67 (0.00)	35.68 (0.02)***	0.85 (0.01)
Escape	3.12 (1.18)	2.28 (1.04)	3.13 (1.05)	2.54 (1.10)	5.24 (0.01)*	151.22 (0.09)***	4.78 (0.00)*
Self-status	1.95 (0.96)	1.60 (0.75)	1.99 (0.93)	1.79 (0.95)	6.52 (0.00)*	33.30 (0.02)***	2.41 (0.00)

*p < 0.05, **p < 0.01, ***p < 0.001.

### 3.2 Associations between social media use motives and SMD symptom severity

As shown in Table 3, all social media use motives were significantly associated with the two SMD subscales Heavy Involvement and Negative Consequences. However, the associations with the motives Social Maintenance, Information and Skills, and Entertainment were all below medium size (i.e., ≤0.30). Furthermore, the strength of the associations varied between Heavy Involvement and Negative Consequences for some motives. The motives Social Maintenance and Entertainment exhibited stronger associations with Heavy Involvement than with Negative Consequences, whereas the motives Social Compensation and Escape had stronger associations with Negative Consequences than with Heavy Involvement. All social media use motives had significant associations with each other. However, the strength of the associations varied between 0.08 (Information and Skill with Self-status) and 0.40 (Entertainment with Escape). Of note was that the two motives related to social connection – Social Maintenance and Social Compensation – were only weakly associated (0.18).

**Table 3:** Results of bivariate correlations and regression analyses, investigating associations between motives and the two types of addictive use of social media (i.e., Heavy Involvement and Negative Consequences).

	SMD Heavy involvement		SMD Negative Consequences	
Motives	r	*β*	r	*β*
Social maintenance	0.29***	0.11***	0.18***	−0.02
Social compensation	0.34***	0.17***	0.41***	0.18***
Information and skills	0.06*	−0.09***	0.11***	−0.01
Entertainment	0.27***	0.18***	0.18***	−0.03
Escape	0.37***	0.16***	0.57***	0.46***
Self-status	0.42***	0.27***	0.43***	0.24***
Explained variance (Adj. *R^2^*)		0.31		0.42

The results of the regression analyses (see Table 3) showed that the motives explained 31% of the variance in Heavy Involvement, with all motives contributing independently. However, the motive Information and Skills had a small negative effect in the regression analyses (i.e., higher levels being related to lower levels of Heavy Involvement). For Negative Consequences, the motives explained 43% of the variance, with only the motives Social Compensation, Escape and Self-status remaining significant. The motive Escape had the strongest effect, but it should be noted that Escape is also included as one of the criteria for Negative Consequences. When the item Escape was excluded from the measure Negative Consequences, the motives Social Compensation, Escape, and Self-status remained significant. Altogether, these three motives explained 32% of the variance in Negative Consequences.

### 3.3 Associations between social media use motives and SMD diagnosis

**Figure 1:**
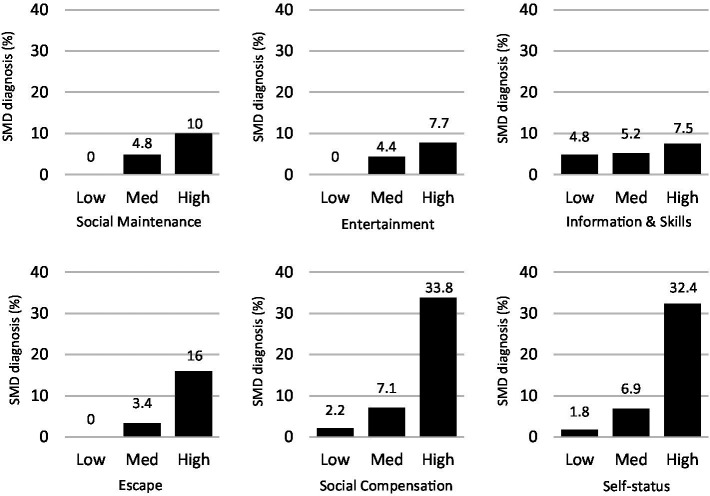
The proportion of individuals meeting the full symptom criteria for SMD diagnosis (≥5 symptoms) among individuals with low, medium, or high levels of each one of the six motives.

Next, we investigated to what extent high levels of each one of the six motives were associated with an increased risk of meeting the full symptom criteria for SMD (i.e., ≥5 symptoms). The results (see Figure 1) showed that individuals with high levels were at a slightly increased risk of meeting the criteria for SMD compared to those with low or medium levels for the motives Social Maintenance (OR = 2.30 [CI 95%; 1.58–3.34]), Entertainment (OR = 1.84 [CI 95%; 1.21–2.82]), and Information and Skills (OR = 1.48 [CI 95%; 1.01–2.17]). However, individuals reporting high levels of the motives Social Compensation (OR = 9.20 [CI 95%; 5.49–15.42]), Self-status (OR = 9.24 [CI 95%; 5.79–14.74]), or Escape (OR = 6.38 [CI 95%; 4.28–9.50]) were much more likely to meet the criteria for SMD compared to those with low or medium levels. As displayed in Figure 1, more than 30% of the participants reporting high levels of the motives Social compensation or Self-status met the criteria for SMD compared to only 2.2 versus 1.8% for those reporting low levels for these motives.

### 3.4 Effects of age and sex for SMD and moderation analyses

A total of 118 individuals (6.2%) met the full symptom criteria for SMD (i.e., ≥5 symptoms). The rate of SMD was significantly higher for females (8.3% for high school students and 8.0% for university students) compared to males (1.1% for high school students and 5.0% for university students), *χ^2^* = 20.00, *p* < 0.001, *V* = 0.11. However, no significant difference was found between high school students (5.8%) and university students (6.8%), *χ^2^* = 0.75, ns, *V* = 0.02.

The moderation analyses revealed that the two motives Social Compensation and Self-status were more strongly associated with Negative Consequences for males (*r* = 0.49 and 0.51) than for females (*r* = 0.36 and 0.37). However, the effects were relatively small, *β* = 0.07 and 0.08, and no significant moderation effects of age group (i.e., high school students versus university students) were found after controlling for multiple comparisons.

## 4 Discussion

The overall aim of the present study was to investigate motives for using social media and associations with SMD symptom severity and SMD diagnosis in adolescents and young adults. Our main findings were that the most prevalent social media use motives were Social Maintenance and Information and Skills, and the least common were Social Compensation and Self-status. Only small age and sex differences were found for the six social media use motives, except for females reporting higher levels of Escape compared to males. A total of 6.3% of the sample met the full symptom criteria for SMD. Females (8%) had higher prevalence rates for SMD compared to males (2.9%), but the prevalence rates did not differ between high school students and university students. The motives Social Compensation, Escape and Self-status had the strongest associations with both SMD diagnosis and SMD symptom severity, accounting for a large portion of the variance in both Heavy Involvement and Negative Consequences. Moderation analyses revealed that the motives Social Compensation and Self-status were more strongly associated with addictive social media use for males than for females. However, no significant moderation effects were found for age.

### 4.1 Prevalence of social media use motives

With regard to how common different social media use motives are, our findings are in line with previous research identifying both Entertainment (Alhabash and Ma, 2017; Süral et al., 2019; Stockdale and Coyne, 2020) and Information and Skills (Brailovskaia et al., 2020; Kircaburun et al., 2020) as common motives. Regarding social connection, the present results showed that it is of great importance to distinguish between Social Maintenance (i.e., maintaining existing relations), Social Compensation (i.e., making new acquaintances) and Self-status (i.e., socializing in order to gain popularity), with the former being the third *most* common motive and the latter two being the two *least* common motives in the present study. The correlation analyses also showed that associations between the motives Social Maintenance and Social Compensation were as low as 0.18. Many previous studies have failed to make a distinction between different types of social interaction motives, but the results of the present study are in line with findings from two previous studies (Süral et al., 2019; Kircaburun et al., 2020). As further discussed below, it is important to note that, although Social Compensation and Self-status are uncommon motives, this does not mean they are unimportant, as they show the strongest associations with addictive social media use.

### 4.2 Age differences

Regarding age differences in the social media use motives, the present study found only negligible to small effects. This is in line with several previous studies finding no effects, or significant but very small effects, of age on social media use motives (Brailovskaia et al., 2020; Kircaburun et al., 2020; Stockdale and Coyne, 2020). As the same results were also found when using longitudinal data and focusing on within-person changes (Stockdale and Coyne, 2020), this might indicate that, although social connections might change quite dramatically during the transition from high school to university, this does not necessarily change the motives for making social connections online (Stockdale and Coyne, 2020). Moreover, reliance on different types of digital media as an escape may be a behavior that is learned relatively early during childhood and that then remains stable within individuals, at least across adolescence and early adulthood, when many individuals are not aware of alternative coping mechanisms. It should also be noted that the studies mentioned above investigated young adult samples or, as in the present study, both adolescents and young adults. It is possible that age differences in social media use motives appear small or non-existent across late adolescence and into young adulthood, whereas they might be larger both earlier and later in life. Orben et al. (2022) investigated social media screen time and life satisfaction in a large sample (*n* = 84,000) spanning in age from 10 to 80 years. Results showed that associations were weakest in older adolescence and emerging adulthood (i.e., the ages included in the present study) and stronger both in early adolescence and in later adulthood. Although this study did not specifically investigate social media motives, the results could be taken to indicate that it is important for future studies to explore effects of age for social media motives in samples spanning in age already from preadolescence to later adulthood.

### 4.3 Sex differences

Regarding sex differences, our findings are in line with those of several previous studies showing that females have higher SMD symptom severity and higher rates of SMD diagnosis compared to males (review by Su et al., 2020). Of more importance to the research questions addressed here, we found a medium effect for the motive Escape, with females reporting higher rates than males. Females also reported higher levels for all the other motives except for Social Compensation, but these effects were negligible to small. This is in line with previous research, in which small effect sizes have commonly been observed when examining sex differences in social media use motives (e.g., Horzum, 2016; Kircaburun et al., 2020).

Regarding the finding that females rated the motive Escape higher than males, this is not in line with the study by Brailovskaia et al. (2020), which failed to find any sex differences for this motive. However, previous research has shown that compared to males, females are more inclined to use emotional coping mechanisms when facing distress, which includes withdrawal, self-criticism, self-distraction, and seeking understanding and sympathy from others (e.g., Matud et al., 2020; Graves et al., 2021). Using social media as an escape may offer a way for females to adopt this coping style, because it can provide support from online friends, but it could also increase withdrawal and self-criticism. To gain further insight, it should be important for future research to better distinguish between escaping offline relations to find online support (i.e., an active coping strategy) and escape as an avoidance strategy that could result in social isolation (i.e., a passive coping strategy) and possibly a reinforcement of existing negative feelings.

### 4.4 Association between motives and SMD symptom severity and SMD diagnosis

The motives Escape, Self-status and Social Compensation showed the strongest associations with SMD symptom severity. These findings are in line with previous studies suggesting that, compared to other motives, these motives are more strongly related to both SMD and mental health problems (Süral et al., 2019; Brailovskaia et al., 2020; Kircaburun et al., 2020; Schivinski et al., 2020). Moreover, individuals who use social media when they are bored or as a way of passing time – behaviors that capture aspects of the Escape motive – show higher scores on measures of problematic social media use (Süral et al., 2019; Kircaburun et al., 2020). What the present study adds is that these three symptoms are also strongly associated with SMD as a categorical disorder. If fact, individuals who reported high levels of the motives Self-status and Social Compensation were almost 10 times more likely to meet the full symptom criteria for SMD compared to those who reported low or medium levels of these two motives. This could mean that these motives may be important risk factors for SMD and, thus, also important to address when developing intervention programs for addictive social media use.

Different explanations are required to understand why the motives Social Compensation, Escape and Self-status appear to be more problematic. According to the Social Compensation Hypothesis (Valkenburg et al., 2005), individuals with high social needs, and at the same time low social competences, use social media to compensate for difficulties making friends offline, which could then lead to increased social problems and addictive social media use. Similarly, Wegmann and Brand (2019) presented the “fear-driven/compensation-seeking hypothesis,” which states that individuals with low social competence and high social anxiety can develop problematic social media use based on negative reinforcement mechanisms (i.e., reductions in fear of social isolation and fear of missing out). The same authors have also presented the “reward-driven hypothesis,” which can explain why the motive Self-status was also strongly associated with addictive social media use. According to this hypothesis, the need for popularity and self-presentation leads people to use social media, and this behavior is increased through positive reinforcement mechanisms (i.e., satisfying the need to belong and the experiences of gratification). However, relying on positive feedback via social media to boost one’s self-esteem can create a negative cycle, where individuals feel a constant need to seek external validation through social media. Previous research has shown that it is primarily upward comparison rather than downward comparison (i.e., comparison to someone who is perceived to be better rather than worse than oneself) that is most strongly related to addictive use of social media as well as to mental health problems such as depression and anxiety (e.g., Gomez et al., 2022).

Regarding the social media motive Escape, high levels of this motive could suggest that the individual is using an inefficient cooping strategy for dealing with negative feelings (e.g., Brailovskaia et al., 2020; Sun and Zhang, 2021). It has been suggested (e.g., Griffiths, 2013) that relying on social networking sites to manage negative emotions establishes a behavior pattern where users become reliant on these platforms to alleviate distress. As this cycle repeats and intensifies, individuals spend more time on social networking sites and find other activities less fulfilling. This reliance on social media to escape feelings or problems can further amplify the negative consequences of social media use, such as neglecting offline responsibilities and not addressing real-life problems. Even if social media are used to actively seek support, research has shown that this may also have negative effects. For example, Cavazos-Rehg et al. (2017) found that 25% of posts on a social media platform provided potentially harmful advice (e.g., advising how to engage in self-harm). In addition, so-called “echo chambers” on social media can create a sense of belonging, but also reinforce preexisting beliefs in a negative way (Cinelli et al., 2021).

It should also be noted that the results of the regression models showed that all motives remained significant predictors of Heavy Involvement, whereas only the motives Social Compensation, Escape, and Self-status remained significant predictors of Negative Consequences. Heavy Involvement includes symptoms such as preoccupation, withdrawal, and tolerance. Using social media for any motive should therefore be related to Heavy Involvement, whereas it is expected that only a few motives are related to Negative Consequences. This finding is in line with several previous studies which have concluded that it is important of distinguishing between media use that is highly engaged and passionate and media use that has negative consequences (e.g., Griffiths et al., 2016; Castro-Calvo et al., 2021; Burén et al., 2023b; Infanti et al., 2023).

### 4.5 Moderation effects of age and sex

Finally, the present study added new information by investigating the moderating effect of sex and age on the associations between the different social media use motives and SMD symptom severity. As such moderation effects have not been investigated previously, a comparison with previous findings is not possible. However, our finding of only two small moderating effects for sex are in line with previous reviews showing no or few moderating effects of sex on the association between social media use and body dissatisfaction (Holland and Tiggemann, 2016). In addition, Jarman et al. (2021) found that their relatively complex model linking social media use motives to addictive use, social media intensity, body satisfaction and well-being was equivalent for males and females. Thus, even though females reported higher levels of SMD symptom severity as well as somewhat higher levels of most social media use motives, the association between motives and addictive use was largely similar for males and females.

### 4.6 Strengths and limitations and future directions

The present study had several strengths, such as a large and diverse sample of both adolescents and young adults, and the use of a psychometrically valid measures to assess all nine criteria for SMD. We also distinguished between two types of addictive social media use (i.e., Heavy Involvement and Negative Consequences). This should be seen as an advantage, given that previous studies have shown that symptoms of negative consequences generally show higher sensitivity in relation to SMD diagnosis (Burén et al., 2021), as well as stronger associations with mental health problems (e.g., Wichstrøm et al., 2019; Burén et al., 2021).

Regarding limitations, it should first be noted that the cross-sectional design of the study limits our ability to infer causality. Associations are most likely bidirectional, with social media use motives influencing SMD symptom severity, but also vice versa. For example, if one uses social media to find new friends (i.e., Social Compensation), this may result in increased social difficulties, further exacerbating the need for social compensation. Consequently, our findings should be replicated in longitudinal studies to study the direction of effects. Second, despite our efforts to collect data from various high schools and universities across Sweden, there remains a possibility that our sample was not fully representative, as only students who attended classes on the day of our visit could be included. Third, motives for social media use were not assessed using an established scale. However, we conducted a thorough literature review as well as a small interview study (*n* = 8) to determine what social media use motives to include in the survey. In addition, the PCA that was conducted to investigate the factor structure indicated that each item loaded on its intended factor. Nonetheless, there remains a risk that this measure may not comprehensively and accurately capture the full range of social media use motives. For example, although previous studies have shown that appearance-based motives (i.e., “to compare how I look with others”) are strongly associated with the Self-status motive (e.g., Rodgers et al., 2021), in future studies it would be interesting to include appearance as a separate motive.

## 5 Conclusion

The main conclusion of the present study was that it was primarily the three motives Social Compensation, Self-status, and Escape that were associated with SMD symptom severity and increased risk of SMD diagnosis. Using social media for maintaining social relations was, however, only weakly associated with SMD. This emphasizes the need to not focus on social interaction in general, but to distinguish between using social media to interact with already well-known acquaintances and using them to compensate for a lack of offline friends. As the effects of age and sex were generally small, these findings apply to both males and females, as well as to adolescents and young adults. Our finding that social media use motives explained a large proportion of the variance in SMD symptom severity could be taken to suggest that the role of motives should be addressed in prevention and intervention efforts targeting problematic social media use. It should also be considered important for future research to assess in more detail what activities that individuals are engaged in when, for example, using social media to increase their status or to escape negative feelings. Previous research has shown that some social media content/behaviors (e.g., creating and sharing nude pictures online; Gassó et al., 2019) are much more strongly associated with mental health problems than are others (e.g., viewing body-positive content; Vandenbosch et al., 2022). It has also been shown that individuals who use social media for entertainment purposes give more “likes” and “dislikes” on social media (Khan, 2017) and show higher social media intensity (Alhabash and Ma, 2017). Moreover, individuals with strong Self-status motives share more information online (Thompson et al., 2020) and use photo-based platforms more frequently (Jarman et al., 2021). Finally, there is a strong need for longitudinal studies within this area of research as the primary motives may change across time. As argued by for example Wood and Griffiths (2007), filling a social void may be the primary motivate at the start. The primary motive may later switch to escaping negative emotions and problems caused by intense media use, creating a vicious circle over time. Conclusively, the results of the present study show that motives are important for a better understanding of social media disorder. However, further studies investigating the links between social media use motives, content, addictive social media use, and mental health problems would be of value.

## Data availability statement

The raw data supporting the conclusions of this article will be made available by the authors, without undue reservation.

## Ethics statement

The requirement of ethical approval was waived by the Swedish Ethical Review Authority for the studies involving humans in accordance with the local legislation and institutional requirements. The study only included anonymous survey data. Thus, it was not possible to link the data to any specific person. The studies were conducted in accordance with the local legislation and institutional requirements. The ethics committee/institutional review board also waived the requirement of written informed consent for participation from the participants or the participants' legal guardians/next of kin because in accordance with the regulations established by the Swedish Ethical Review Authority, parental consent was not needed given that all participants were at least 15 years old. However, written informed consent was obtained from the adolescents themselves.

## Author contributions

LT: Conceptualization, Data curation, Formal analysis, Funding acquisition, Investigation, Methodology, Supervision, Writing – original draft, Writing – review & editing. MA: Data curation, Writing – original draft, Writing – review & editing. AR: Data curation, Writing – original draft, Writing – review & editing. JB: Conceptualization, Data curation, Writing – original draft, Writing – review & editing. SN: Conceptualization, Data curation, Writing – original draft, Writing – review & editing.
